# Genome sequencing of *Pasteurella multocida* strains isolated from Jinding duck (*Anas platyrhynchos*) in Bangladesh

**DOI:** 10.1128/mra.01150-25

**Published:** 2026-02-27

**Authors:** Md. Alimul Islam, Md. Enamul Haque, Md. Iftehimul, Mohammad Sadekuzzaman, Tanbin Rubaiya Islam, Md. Sojon Miah, Sajedul Hayat, Susmita Karmakar, Muhammad Tofazzal Hossain

**Affiliations:** 1 Department of Microbiology and Hygiene, Bangladesh Agricultural University 54492, Mymensingh, Bangladesh; 2 Institute of Biotechnology, Bangladesh Agricultural University, Mymensingh, Bangladesh; 3 Central Disease Investigation Laboratory, Department of Livestock Services 206809 , Dhaka, Bangladesh; Indiana University, Bloomington, Bloomington, Indiana, USA

**Keywords:** *P. multocida* DC3 and DC4, fowl cholera, Jinding duck

## Abstract

Fowl cholera, a highly transmissible disease in ducks, is caused by *Pasteurella multocida*; we report the two strains of *P. multocida*, DC3 and DC4. The assembled genome sizes were ~2.49 Mbp (DC3) and ~2.5 Mbp (DC4) with sequence depth coverage 1,043 and 899×, respectively. The G+C contents were 40.45% and 40.38%, respectively.

## ANNOUNCEMENT

*Pasteurella multocida,* the causative agent of fowl cholera, is highly prevalent in ducks in Bangladesh, with 20–40% infection rates in outbreaks and 30–80% flock mortality (1, 2), posing a major threat to duck production and rural livelihoods. Here, we announce the genome sequence of *P. multocida* strains (DC3 and DC4) isolated from the fowl cholera suspected Jinding duck (*Anas platyrhynchos*) heart samples in Kishoreganj and Netrokona, Bangladesh. The collected samples were incubated overnight at 37°C in Luria-Bertani (LB) broth (Oxoid, Thermo Fisher Scientific, UK). A loopful of the inoculum from the LB culture was streaked onto blood agar base (Oxoid, Thermo Fisher Scientific, UK) enriched with 5% sheep blood and incubated at 37°C for 24–48 h. Total DNA was extracted with the Wizard Genomic DNA Purification Kit (Promega Corporation, Madison, WI) in accordance with the manufacturer’s instructions, and sequencing libraries were made with the Illumina DNA Prep Library Preparation Kit (Illumina, Inc., San Diego, CA); sequencing was performed on Illumina NovaSeq 6000. Sequencing of strain DC3 generated 8,686,239 reads, while strain DC4 generated 7,523,898 reads with an average read length of 150 bp. FastQC v0.12.1 (3) have been used to check the quality of sequence reads and filtered using Fastp v0.24.0 (4)*,* then *de novo* assembled with SKESA v. 2.2 (5), and the post quality resulting assemblies were checked with QUAST v5.3.0 (6). To assess assembly accuracy, genome completeness and contamination were determined with CheckM v1.2.3 (7), and average nucleotide identity (ANI) values were determined through the OrthoANI algorithm implemented in EZBioCloud (8) to a reference (accession: GCF_002073255.2). Genome annotation of DC3 and DC4 was performed using the NCBI Prokaryotic Genome Annotation Pipeline (PGAP) v6.9 (9). Functional characterization included identification of antibiotic resistance genes (ARGs) with ResFinder (10), plasmid sequences with PlasmidFinder (11), and virulence factors (VFGs) through VFDB (12), all executed via ABRicate v0.8.13. Pathogenic potential was further predicted with PathogenFinder v1.1 (13). Mobile genetic elements (MGEs) were screened using ICEFinder v2.0 (14). All analyses were performed using default settings, unless otherwise specified.

The assembled genome of *P. multocida* strains comprises 2,496,320 bp (DC3) and 2,508,432 bp (DC4) spanning over contigs 72 and 47, with genome coverage 1,043 and 899×. The GC content was 40.45 (DC3) and 40.38% (DC4), respectively. Assembly quality metrics included L50 values of 9 (DC3) and 4 (DC4), with corresponding N50 values of 76,000 and 163,637 bp, respectively. A summary of genome characteristics is provided in Table 1.

**TABLE 1:** Information regarding genome sequence of *P. multocida* strains isolated from the fowl cholera suspected Jinding duck (*Anas platyrhynchos*) in Bangladesh

Parameter	*Pasteurella multocida* DC3	*Pasteurella multocida* DC4
BioSample accession	SAMN47638443	SAMN47638444
GenBank accession	GCA_049347985	GCA_049348075
SRA accession	SRS24546956	SRS24546957
Nucleotide accession	DBHLZS000000000	DBHLZX000000000
Genes (total)	2,403	2,418
CDSs (total)	2,343	2,358
Genes (coding)	2,322	2,335
CDSs (with protein)	2,322	2,335
Genes (RNA)	60	60
rRNAs	2, 2, 1 (5S, 16S, 23S)	2, 2, 1 (5S, 16S, 23S)
Complete rRNAs	1, 1 (5S, 23S)	1, 1 (5S, 23S)
Partial rRNAs	1, 2 (5S, 16S)	1, 2 (5S, 16S)
tRNAs	51	51
ncRNAs	4	4
Pseudogenes (total)	21	23
CDSs (without protein)	21	23
Pseudogenes (ambiguous residues)	0 of 21	0 of 23
Pseudogenes (frameshifted)	8 of 21	9 of 23
Pseudogenes (incomplete)	8 of 21	9 of 23
Pseudogenes (internal stop)	9 of 21	10 of 23
Pseudogenes (multiple problems)	3 of 21	4 of 23
CheckM completeness	100	100
CheckM contamination	3.8	4.8
Genome quality	Good	Good
Average nucleotide identity (ANI)	98.67	98.59
Plasmid	1	1
Pathogenicity	83.4%	82.6%
AMR genes	5	5
Virulence genes	3	3
MGEs	4	3

## Data Availability

All raw genome sequence data of *Pasteurella multocida* were submitted to NCBI, GenBank, and SRA under BioProject number PRJNA514245.
